# Fatigue Crack Growth Rate Description of RF-Plasma-Sprayed Refractory Metals and Alloys

**DOI:** 10.3390/ma16041713

**Published:** 2023-02-18

**Authors:** Ondrej Kovarik, Jan Cizek, Jakub Klecka

**Affiliations:** 1 Faculty of Nuclear Sciences and Physical Engineering, Czech Technical University in Prague, Trojanova 13, 120 00 Prague 2, Czech Republic; 2 Institute of Plasma Physics of the Czech Academy of Sciences, Za Slovankou 1782/3, 182 00 Prague 8, Czech Republic

**Keywords:** fatigue crack growth, analytical model, refractory materials, RF plasma spray

## Abstract

A fitting method capable of describing the fatigue crack growth rate (FCGR) data in all stages of crack propagation by a simple Forman-style analytical formula was developed. To demonstrate its robustness, this method was used to quantify the fracture behavior of RF-plasma-sprayed W, Mo, W-Mo composite, and four selected Ni-based tungsten heavy alloys (WHA). The fitted FCGR parameters categorized the studied materials into two distinct sets. W, Mo, and W-Mo composite deposits made from inherently brittle refractory metals that contained a range of defects inherent to plasma spray process represented the first class. This class was characterized by low fracture toughness and a relatively wide range of fatigue crack growth thresholds. The second class of materials was represented by WHA. Here, the deposit defects were suppressed by liquid state diffusion that formed a typical WHA structure consisting of a Ni-rich matrix and large spherical W reinforcement particles. The WHA generally showed higher fatigue crack growth thresholds, but differed in fracture toughness values based on the W particle concentrations. The obtained fracture mechanical data represent a reference dataset of plasma-sprayed refractory materials, and their classification into groups clearly demonstrates the capabilities of the developed method to capture a wide range of different types of FCGR behavior.

## 1. Introduction

Tungsten has the highest melting temperature of all metals, very high density, and acceptable radioactive dose rate after its activation. These properties qualify it for use in heat shields of thermonuclear devices. Unfortunately, at the same time, tungsten suffers from low fracture toughness below its transition temperature (∼400–800 ${}^{\circ }$C [1,2]), in-service recrystallization, and an easily triggered oxidation. As well as other high-Z refractories, such as Mo, W further suffers from surface fuzz due to He${}^{+}$ irradiation [3]. Last, bulk tungsten machining is very complicated.

Given all these, W alloys are often considered as an alternative to pure W, especially in cases where mechanical loading is important. Of these, a very interesting family are the tungsten heavy alloys (WHAs), composite materials with W particles embedded in a ductile Ni-based matrix. WHAs seem to overcome most of the deficiencies of pure tungsten, and are considered as candidate materials for the first wall of future fusion devices [4]. For such an application, new manufacturing methods that offer an alternative to conventional powder metallurgy are being investigated. Thermal spray represents one of them, capable of covering relatively large and geometrically complex shapes [5] or producing multiphase, multilayered, or graded coatings [6,7]. From all thermal spray methods, this paper focuses on plasma spray due to its sufficient heat transfer to the feedstock material to enable the WHA alloy formation. Unfortunately, for a typical atmospheric plasma spray process, oxidation represents a major problem, and either vacuum systems [8,9] or systems using gas shrouding [10,11] need to be used. As much as the in-flight material oxidation is effectively suppressed this way, the oxygen originally present in the feedstock material [8] still represents a significant problem that needs a solution. In 2011, Wang et al. [12] demonstrated that a WHA deposit can be plasma-sprayed. The authors were able to obtain deposits of nominal composition W-3.5Ni-1.5Fe by plasma spraying from spray-dried powders agglomerated from a fine feedstock and showed that the controlled Ar atmosphere inside the deposition chamber can efficiently eliminate the oxidation. Annealing of the coating was still necessary to achieve the optimal two-phase alloy composition consisting of pure tungsten particles in a Ni-rich matrix, though. In our previous work [13], this annealing step was avoided by maintaining the substrate temperature above 1100 ${}^{\circ }$C during the spraying, significantly simplifying the manufacturing process.

The major limitation of WHA is the low melting temperature of the Ni-based matrix that lies slightly above 1500 ${}^{\circ }$C. In our recent experiments, we have deposited a Mo-20W (wt%) composite. This material is significantly more heat-resistant than WHA, but also offers two other advantages: Mo is relatively easy to deposit by RF [9,14] and, further, W shows a complete solubility in Mo. These composites were also included in this paper to show the capability of the fitting method to capture different kinds of brittle materials.

Up to now, only a limited number of papers on the fracture properties of thermally sprayed refractory metals or WHA have been published [8,9,15]. In fact, a complex fracture mechanical properties characterization of this important class of materials accompanied with a thorough mechanical properties characterization is still missing. This study aims to fill this gap by putting forward a complex fracture mechanical investigation from fracture toughness tests (based on the ASTM E1820 standard [16]), fatigue crack growth rate tests (based on the ASTM E647 standard [17]), and stress–strain properties measurement. For all these, a recently developed, unified, simple rectangular bar geometry specimen (EDM-cut from the thick deposits) was used [18], thereby significantly simplifying the sample preparation as well as the testing procedures. The obtained results were complemented with the data published in our earlier studies [9,15,19] and numerically characterized using the new fitting method introduced in this paper. This method was designed to provide a simple description of the fracture behavior of a particular material by its fatigue crack growth threshold $\Delta K_{\mathrm{thr}}$, fracture toughness $K_{\mathrm{IC}}$, as well as Paris-type coefficient *D* and exponent *p*. A direct comparison of the fracture properties of Mo and W deposits, Mo-W composite, and several tungsten heavy alloys was thus possible.

## 2. Materials and Methods

### 2.1. Deposition

The materials deposition was performed using TekSpray15 (Tekna, Sherbrooke, Canada) radio frequency inductively-coupled plasma system (RF-ICP). The plasma was generated at 15 kW of plate power (the maximum of the system) from Ar-H${}_{2}$ gas mixture (central gas flow: 10 slpm Ar, sheath gas flow: 35 slpm Ar + 3.4 slpm H${}_{2}$), providing the protective (and partially reducing) atmosphere.

Two types of graphite substrates were used: cylindrical discs (d=60mm, h=10mm) mounted onto a non-cooled holder and thin strips ($50\times 20\times 3{\mathrm{mm}}^{3}$) mounted onto an in-house developed water-cooled holder. The deposition onto the discs was performed by a stepwise traversal movement of the holder below the torch (starting at the discs center) at a constant disc revolution of 22 rpm. The combination of the two movements resulted in different torch scanning speeds (and thus different deposition rates) at different distances from the discs centers; this was compensated by different numbers of revolutions spent at each holder position. Contrary to this, the deposition onto the strips was performed in a linear manner, with the samples performing a reciprocating traversal movement under the torch over a 42 mm distance at a scanning speed of 1 mm·s${}^{-1}$.

The used feedstock powders were commercially available metal powders (Table 1). From these powders, five more blends were prepared and deposited (Table 2).

The Mo-20W composition represented a pilot experiment focusing on the W-Mo interface properties in a plasma-sprayed heat-resistant composite. WHA compositions W-10Ni, W-20Ni, and W-65Ni formed the basis for a study of the role of matrix and particles in the failure mode of plasma-sprayed WHA. The two studies on Mo-20Ni composite and WHA will be published as separate papers.

Due to the limited available torch power (15 kW), the used particle size ranges were significantly smaller (10–45 µm) than what is normally used in standard plasma spraying. Some of the powders were pre-treated before their deposition in order to reduce the amount of oxygen adsorbed at the surface (these are indicated by a “(pre)” symbol in Table 2). During this treatment, the powders were held at a constant temperature under a 1.5 slpm flow of Ar-7.5H${}_{2}$ gas mixture: for W-10Ni, W-20Ni, and W-65Ni, this was 2 h at 400 ${}^{\circ }$C, while for Mo, W, and their Mo-20W mixture, this was 20 minutes at 900 ${}^{\circ }$C.

Aside from these, two additional powder metallurgy W-6Ni-3Co (wt.%) reference WHA materials from our previous work [13] were included in this study. Both were prepared by cold isostatic pressing, sintered at 1530 ${}^{\circ }$C, annealed, and quenched. The material denoted as PM-SQ was sintered and quenched, while the material denoted PM-FA was further rotary forged at 400 ${}^{\circ }$C and annealed.

### 2.2. Fatigue Crack Growth Rate

**Figure 1:**
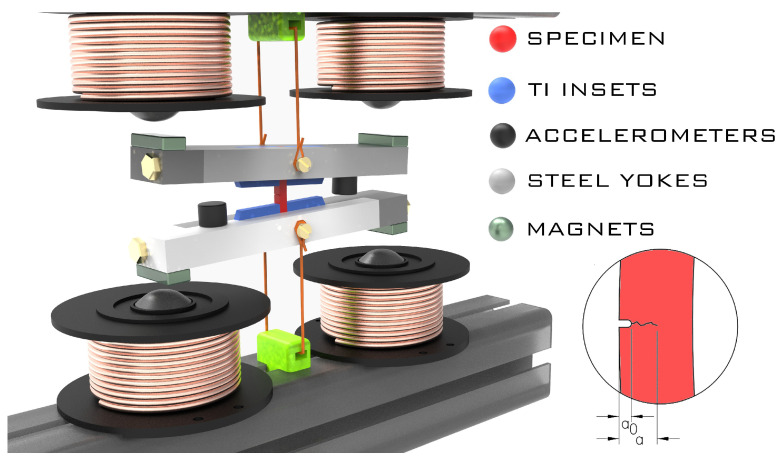
Schematic drawing of the resonance bending fatigue crack growth test apparatus (see [18]).

**Figure 2:**
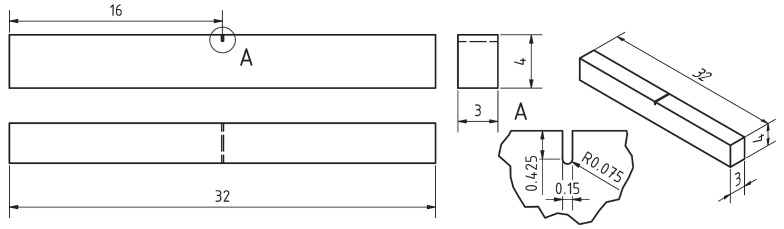
The simple geometry and dimensions (in mm) of the universal specimen used for multiple tests. The notch is present in specimens designated for fracture toughness and fatigue crack growth rate tests, while the stress–strain test specimens are un-notched.

The resonance bending setup used for the FCGR testing as well as for a fatigue pre-cracking of samples designated for fracture toughness testing is shown in Figure 1, while the used specimen geometry is shown in Figure 2. A more detailed description of the testing methodology and comparison with standard testing methods can be found in our previous study [19]. The vibrating system is represented by an H-shape assembly defined by two inertial yokes and the specimen. The system is excited to its first bending mode of vibration. The bending moment applied to the specimen is evaluated from the yokes’ acceleration. The resonance nature of the system predetermines the remote stress ratio R∼−1 before a crack initiates. As the fatigue crack grows, *R* decreases to R∼−1.5. The crack length was measured using a differential compliance method developed by Golub [20]. The crack-added compliance of the specimen was determined in a time domain, by fitting the vibration waveform. This method leads to an unprecedented precision of the crack length measurement. This, in turn, enabled employing a so-called rate control mode. In this mode, the specimen loading is controlled by a closed loop so that the prescribed crack growth rate $\frac{da}{dN}$ is maintained. The experiments started with pre-cracking from the notch at $\frac{da}{dN}=10^{-9}$ m·cycle${}^{-1}$, gradually decreasing the crack growth rate so that $\frac{da}{dN}=10^{-11}$ m·cycle${}^{-1}$ was reached at a crack length of a=1.1 mm. The crack growth rate was then increased up to $\frac{da}{dN}=10^{-6}$ m·cycle${}^{-1}$ at a=2 mm.

### 2.3. Mechanical Properties Testing

Mechanical properties of the deposits were assessed in bending using the method of Herbert [21]. This method enables the extraction of uniaxial stress–strain data in tension and compression from a single bend test. Four-point bending (4PB) fixture with the outer span of 27 mm and the inner span of 13.5 mm was used. Using the differential formulas of Maywille and Finnie [22], the measurement evaluates the stress–strain curves from the record of a loading force and strains $\varepsilon_{c}$ and $\varepsilon_{t}$ on the compressive and tensile specimen surfaces:

(1) $$\sigma_{c}=\frac{dM\left(\varepsilon_{c}+\varepsilon_{t}\right)+2M\left(d\varepsilon_{c}+d\varepsilon_{t}\right)}{bh^{2}d\varepsilon_{c}},\;\sigma_{t}=\frac{dM\left(\varepsilon_{c}+\varepsilon_{t}\right)+2M\left(d\varepsilon_{c}+d\varepsilon_{t}\right)}{bh^{2}d\varepsilon_{t}},$$

where $\sigma_{c}$, $\sigma_{t}$ correspond to compressive and tensile stresses, *M* is the applied bending moment, and *h* and *b* represent the specimen width and thickness, respectively. The strains $\varepsilon_{c}$ and $\varepsilon_{t}$ were obtained by fitting the displacement field measured by digital image correlation (DIC) with analytical formula for pure bending. The method is valid up to 5% of the specimen surface strain.

Fracture toughness was measured following the ASTM E1820 standard [16] using the same fixture modified into a three-point bending configuration. The crack length was measured by the DIC technique described in detail in [18]. The fracture toughness specimens were fatigue pre-cracked at $\frac{da}{dN}=10^{-9}$ m·cycle${}^{-1}$. Additionally, specimens with cracks generated during FCGR tests were also used to measure fracture toughness, and the obtained $K_{\mathrm{IC}}$ values were fully comparable with the dedicated fracture toughness specimens.

Both the stress–strain and the fracture toughness tests used the same specimen geometry as the FCGR test. A more detailed description of the methods can be found in our previous paper [18].

### 2.4. Hartmann–Schijve Fit

Many mathematical models can be fitted to the fatigue crack growth data, including the well-known formulas of Paris, Klesnil–Lukas, or different variants of the Forman-type equation. For a more detailed description of these models, the reader is directed to the review paper of Schwalbe [23].

In our paper, the Hartmann–Schijve (H-S) variant [24] of Forman equation was selected for its simplicity and capability to fit all three stages of the fatigue crack growth, i.e., the near-threshold, Paris, and cyclic fracture toughness regimes. The H-S formula already demonstrated its capability to fit the FCGR data from small specimen tests in [25,26,27,28]. The method to fit the H-S model to FCGR data must reflect the asymptotic behavior of the model. The model approaches both the FCGR threshold $\Delta K\to \Delta K_{\mathrm{thr}}$ and cyclic fracture toughness $K_{\max}\to A$. This leaves the model functions undefined outside these limits, complicating the fitting. Two approaches can overcome this deficiency: total least-squares fitting, or inverse function fit. Recently, Schoenherr et al. [29] used the inverse approach to fit the near-threshold stage of the fatigue data with a newly proposed model. In such approach, the high slopes of the $\frac{da}{dN}$ curve at the near-threshold and cyclic fracture toughness boundary transform into constants. In this paper, we follow a similar approach, and use $K_{\max}$ and ΔK as dependent variables of the fit. The fit of the $K_{\max}=K_{\max}\left(\frac{da}{dN}\right)$ function, however, requires the H-S model to be inverted as described below.

The H-S model is based on the Paris-type formula:

(2) $$\frac{da}{dN}=D\Delta \kappa^{p},$$

where Δκ is the Schwalbe’s crack driving force [23] defined as

(3) $$\kappa =\frac{\Delta K-\Delta K_{\mathrm{thr}}}{\sqrt{1-\frac{K_{\max}}{A}}}.$$

Here, *A* is cyclic fracture toughness and $\Delta K_{\mathrm{thr}}$ is the value approached by the stress intensity factor range at the fatigue crack growth rate threshold. Note that this is dissimilar to another threshold value $\Delta K_{\mathrm{th}}$ corresponding to $\frac{da}{dN}=10^{-10}$ m·cycle${}^{-1}$, as defined in ASTM E647 [17]. The conversion from the fitted $\Delta K_{\mathrm{thr}}$ can be evaluated from Equations (5) and (8).

Only positive stress intensity factors, $K_{\max}$, ΔK, and Schwalbe’s crack driving force Δκ, are considered:

(4) $$K_{\max}>0,\Delta K>0,\Delta \kappa >0.$$

For known stress ratio *R* experiments, this yields

(5) $$\Delta K=K_{\max}\left(1-R\right),$$

and Equation (3) therefore transforms to

(6) $$\Delta \kappa =\frac{K_{\max}\left(1-R\right)-\Delta K_{\mathrm{th}}}{\sqrt{1-\frac{K_{\max}}{A}}}.$$

As noted in Equation (4), Δκ must be positive. At the same time, the denominator of Equation (6) must be real. Therefore

(7) $$K_{\max}<A\wedge \Delta K_{\max}>\frac{K_{\mathrm{thr}}}{1-R}$$

The value of Δκ outside the interval described by Equation 7 is undefined, thus the error of the fit cannot be computed for such datapoints. Moreover, the model function has very high gradients near $\Delta K_{\mathrm{thr}}$ and *A*, further complicating the fit.

One solution of the above problem would be fitting the inverse function $K_{\max}=K_{\max}\left(\frac{da}{dN}\right)$ to the experimental data. Fortunately, both Equations (2) and (3) can be easily inverted:

(8) $$\begin{array}{ccc} \Delta \kappa = & {\left(\frac{1}{D}\frac{da}{dN}\right)}^{\frac{1}{m}},\; & \\ K_{\max}= & \frac{\Delta \kappa }{2A\left(R^{2}-2R+1\right)}(\sqrt{4A\left(\Delta K_{\mathrm{thr}}\left(R-1\right)+A\left(R^{2}-2R+1\right)\right)+\kappa^{2}}\\ - & \kappa^{2}+2A\Delta K_{\mathrm{thr}}\left(1-R\right)). \end{array}$$

The domain of the inverse Equation (8), i.e., the range of $\frac{da}{dN}$, is only limited by the part of Equation (8) below the radical:

(9) $$4A\left(\Delta K_{\mathrm{thr}}\left(1-R\right)-A\left(R^{2}-2R+1\right)\right)<\kappa^{2}.$$

Fortunately, $\Delta K_{\mathrm{thr}}\left(1-R\right)-A\left(R^{2}-2R+1\right)$ is always negative for R=−1. For R=0.1, it is necessary to have $A\geq 1.1\Delta K_{\mathrm{thr}}$, while for R<0.8, it is necessary to have $A\geq 5\Delta K_{\mathrm{thr}}$. For even higher *R* values, imposing bounds on either $\Delta K_{\mathrm{thr}}$ or *A* may be desirable.

Fitting Equation (8) to experimental data can be performed by any nonlinear iterative technique. The bounded fitting desirable for positive stress ratios can be carried out through, e.g., the trust region reflective algorithm ([30], used in the methodology presented here).

The described fitting procedure was implemented as a Matlab (The MathWorks Inc., Natick, MA, USA) script that enables batch fitting and plotting of FCGR data. The script as well as its documentation are accessible as supplementary data files of this paper.

### 2.5. Metallography

In order to illustrate the microstructure of the investigated materials, pseudo-3D figures were prepared using electron channeling contrast (ECCI) images captured using JEOL IT-500HR (JEOL, Tokyo, Japan) scanning electron microscope (SEM). The metallographic cross-sections were prepared using standard techniques with a final polishing stage of 8 h polishing in colloidal silica using Vibromet (Buehler, Lake Bluff, IL, USA) vibratory polisher. Chemical composition was studied by Octane Elite (Ametek EDAX, Pleasanton, CA, USA) energy dispersive X-ray analysis (EDX) system.

## 3. Results and Discussion

### 3.1. Microstructure

**Figure 3:**
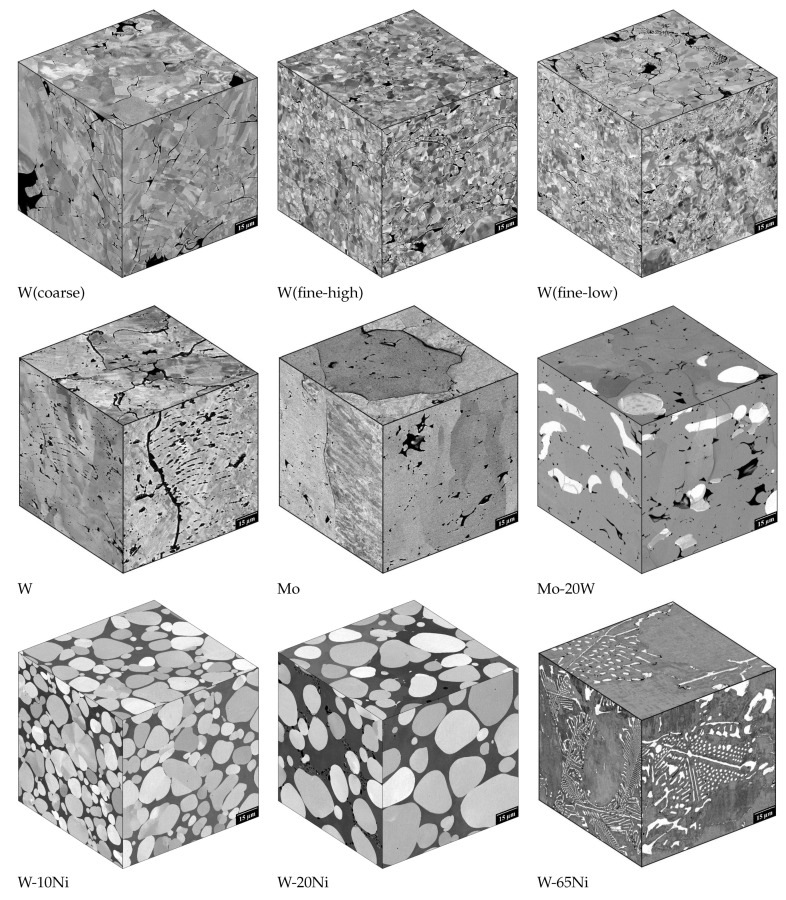
Pseudo-3D microstructures of the investigated refractory materials.

**Figure 4:**
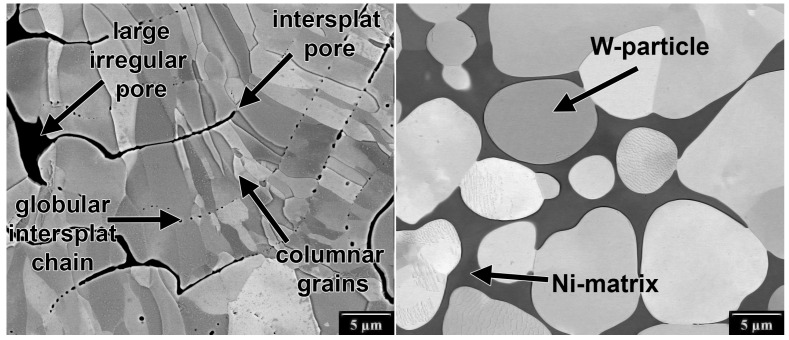
Typical micromorphological features seen in Mo and W deposits illustrated using W (coarse) deposit (**left**), and typical features of WHA illustrated using W-20Ni deposit (**right**).

The microstructures of W, Mo, W-20Mo, W-10Ni, W-20Ni, and W-65Ni are presented in Figure 3. The most typical defects and other micromorphological features are shown in Figure 4.

The W and Mo deposits showed small globular pores chained along splat boundaries, narrow inter-splat pores, and larger irregular pores caused by unmelted or resolidified particles. The materials consisted of columnar grains spanning several layers of splats (for more details see [9,19]). The W particles in the Mo-20Ni deposit indicated proper W particles flattening without any significant splashing.

The microstructure of the W-10Ni and W-20Ni WHA deposits showed a fully developed composite microstructure with spherical pure W particles embedded in the binder matrix formed by a saturated solid solution of W in Ni (see Figure 4 and [13]). In contrast to the pure Mo and W deposits, the splat boundaries and pores were missing in the WHA, yielding them almost defect-free. The homogeneous composition of the matrix indicated that a saturated W solution in Ni was reached. At the same time, the spherical shape of the particles together with their size indicated that they were formed during the cooling phase, driven by the solubility decrease of W in Ni. In the W-65Ni deposit, a fine, pro-eutectic W phase embedded in the matrix was observed, indicating that a hyper-eutectoid W concentration was reached. The corresponding Ni contents determined using energy-dispersive X-ray analysis were approximately 8 wt. % for W-10Ni and W-10Ni* deposits, 15 wt. % for W-20Ni deposit and 48 wt. % for W-65Ni deposit, indicating a selective (Ni-deficient) deposition efficiency.

### 3.2. Fracture Properties

**Figure 5:**
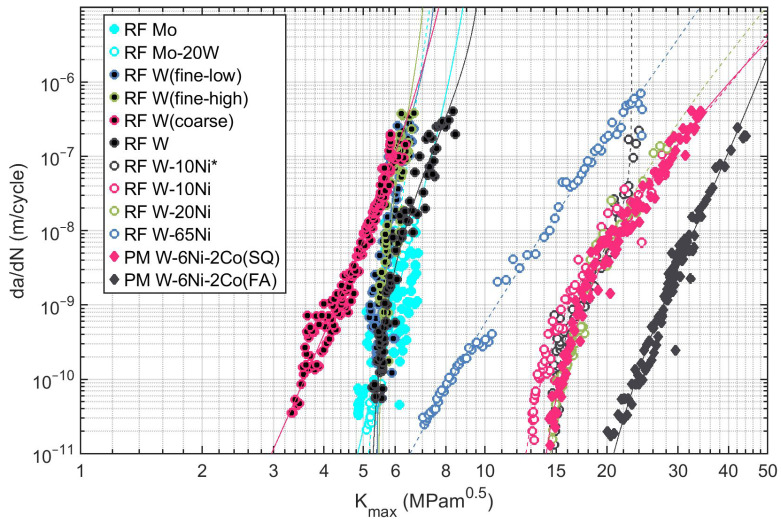
Fatigue crack growth data of the investigated refractory materials.

The results of the FCGR tests are summarized in Figure 5, where the maximum value of the stress intensity factor $K_{\max}$ seen during one loading cycle is plotted as the independent variable. For several reasons, $K_{\max}$ characterizes well the crack driving force for the used negative stress ratios R<−1: obviously, when the Schwalbe crack driving force from Equation (3) is considered, $K_{\max}$ in the denominator defines the FCGR curve when $K_{\max}$ approaches the cyclic fracture toughness *A*. The nominator $\Delta K-\Delta K_{\mathrm{thr}}$ should, however, be treated carefully under the used negative stress ratio. There are many suggestions for the crack driving force under negative stress ratios based on $K_{\max}$ instead of ΔK which usually include the effect of crack tip compressive stress $\sigma_{\mathrm{tip}}$ as discussed, e.g., by Benz and Sandner in [31]. In the used specimen bending configuration, however, $\sigma_{\mathrm{tip}}$ is relatively low at the start of the FCGR experiment at a=1.1mm as the contacting crack faces already take a significant part of the load (note that at this point, the crack extends from the notch, i.e., from $a_{0}∼0.5\;$mm up to a∼1.1mm). Further decreases in $\sigma_{\mathrm{tip}}$ take place and $\sigma_{\mathrm{tip}}$ approaches zero at a∼2.2mm. Neglecting the effect of $\sigma_{\mathrm{tip}}$, the recommendation of the ASTM standard E647 [17] can be adopted and $\Delta K=K_{\max}$ can be safely assumed. This assumption is equivalent to setting R=0 in Equation (8), simplifying the problem significantly. Note, however, that the fitting procedure considers non-zero *R* values in order to enable fitting of positive stress ratio data.

In general, the data in Figure 5 show two distinct groups of the FCGR curves. W, Mo, and Mo-20W have a rather low fatigue crack growth resistance and—in most cases—very steep FCGR curves. This is caused by the inherent brittleness of W and Mo, amplified by the micromorphology of the deposit containing weak grain boundaries, splat interfaces, and porosity. The formation of these defects is caused by the rapid cooling of the deposited splats, driven by the difference of the high melting point of the refractories and the substrate temperature that was lower than 1500 ${}^{\circ }$C.

Contrary to those, the WHAs exhibit nearly half an order of magnitude higher $K_{\max}$ values and less steep FCGR curves. This is given by the ductile nature of the Ni-rich matrix combined with the reinforcement effect of the W particles, and the strong particle–matrix diffusion bonds. Most of the WHA deposits show near-threshold elbow when $K_{\max}$ asymptotically approaches the threshold value $\Delta K_{\mathrm{thr}}$. The exceptions from this rule are the W-65Ni ductile matrix deposit without the reinforcement particles and the forged and annealed PM W-6Ni-3Co (FA) reference.

The high load elbow, where the $K_{\max}$ values asymptotically approach the cyclic fracture toughness *A*, was only observed for the W-10Ni* material. This fatigue crack growth acceleration was promoted by a brittle fracture of the W particles from an untreated powder [15]. For other materials, this kind of asymptotic behavior was not observed as it probably happened in the $\frac{da}{dN}$ range not captured in our experiments. Therefore, the value of *A* may not be fitted reliably from such experimental data. Still, the static fracture toughness $K_{\mathrm{IC}}$ may offer an approximation of the cyclic fracture toughness *A*, even though the relation $A=A(K_{\mathrm{IC}})$ is not straightforward. The modeling approach of Roman and Ono [32] suggests that $A∼K_{\mathrm{IC}}$ for materials without significant strain hardening or softening. In our study, this approach is therefore justified for the W, Mo, and Mo-20W materials with limited plasticity. For other, more ductile materials containing Ni with significant cyclic softening, the cyclic fracture toughness *A* may be expected to reach up to two times higher than the static fracture toughness $K_{IC}$ (see [32]). To address this fact, the fracture toughness of both specimens pre-cracked during the FCGR test and specimens pre-cracked at $\frac{da}{dN}=10^{-9}\;$m·cycle${}^{-1}$ was tested. No significant differences were observed, therefore $A=K_{\mathrm{IC}}$ was assumed for all specimens and the parameter *A* of the fit was thus fixed. Note that this approach uses a conservative approximation of *A*, rendering it safe from the engineering point of view.

**Figure 6:**
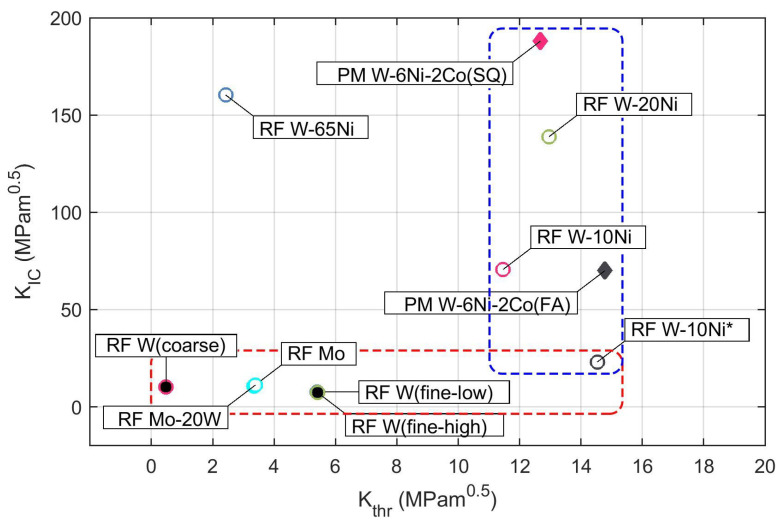
Fatigue crack growth threshold $\Delta K_{\mathrm{thr}}$ and fracture toughness $K_{\mathrm{IC}}$ of the investigated refractory materials.

The coefficients of the H-S fit (Equations (2) and (3)) of the experimental data are provided in Table 3 along with the stress–strain data obtained from the bend tests. Each particular dataset was fitted separately as the deposits differed significantly in terms of the feedstock and the used RF-ICP deposition parameters. In order to summarize the results of the fit, two important engineering characteristics, fatigue crack threshold $\Delta K_{\mathrm{thr}}$ and cyclic fracture toughness *A* (approximated by quasi-static fracture toughness $K_{\mathrm{IC}}$), are plotted in Figure 6.

**Figure 7:**
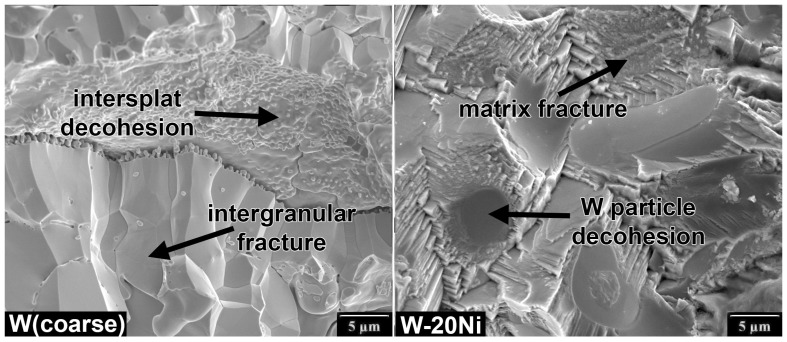
Fracture surfaces of W (coarse) deposit with the worst fracture properties and fracture surface of the W-20Ni WHA with the best fracture properties among all RF-sprayed materials (see Figure 6).

Pure W and Mo as well as Mo-20W lie in a horizontal band of limited fracture toughness, but feature a wide range of $\Delta K_{\mathrm{thr}}$ given by the used feedstock material. Considering the stress–strain properties, these materials can be regarded as brittle, with limited plasticity observed only for the Mo deposit. The fracture properties seem to correlate with strength and modulus, with the best fracture properties obtained for Mo and W from the pre-treated powders, and the worst properties obtained for W from the as-received coarse W powder. The observed FCGR curves for pure W show much lower resistance that the rolled W sheets tested recently by Pillmeier et al. [33], indicating $K_{\mathrm{th}}$ = 14.6–23 MPa·m${}^{0.5}$ and the Paris exponent *m* = 2.6–3. The fracture surface of the RF W (coarse) deposit representing this group of materials with typical plasma spray microstructure is shown in Figure 7. Most of the fracture is intergranular, with a significant contribution by splat decohesion accelerated by the intersplat porosity, which corresponds to the observed low fatigue crack growth resistance.

The studied tungsten heavy alloys, on the other hand, lie in a vertical band in a limited $\Delta K_{\mathrm{thr}}$ range, with $K_{IC}$ increasing with an increasing Ni content. The W-65Ni alloy stands out as it features a very low $\Delta K_{\mathrm{thr}}$, probably due to the lack of strengthening by the W particles, indicated by the lower yield strength $R_{p0.2t}$. On the other hand, the near-threshold behavior of the PM W-6Ni-3Co (FA) strengthened by the rotary forging to $R_{p0.2t}=1596$ MPa showed significant increase in the $\Delta K_{\mathrm{thr}}$ threshold. At the same time, the resulting lack of ductility probably caused the fracture toughness decrease to $K_{IC}=70$ MPa·m${}^{0.5}$. Consistently, pretreating the powder led to a decrease in yield strength $R_{p0.2t}$ and, in turn, to higher fracture toughness as manifested by comparing the W-10Ni* and W-10Ni materials. It is interesting to compare the W-10Ni* FCGR data with the results of Roman and Jinchuk [34], who tested powder metallurgy W-Ni-Fe WHA and obtained a very steep curve with Paris exponent n=12 at *R* = 0.1 at a comparable $K_{\max}$ range. They attributed the high Paris slope to cleavage cracking of the particles. In our case, the W particle cracking took place at high loads, accelerating the crack growth and forming the above-discussed high load elbow. The fitted exponent p=1.54 was, on the other hand, rather low, indicating a progressive change of the fracture mechanism with an increasing amount of cleavage fracture at higher loads. The fatigue fracture surface of RF W-20Ni deposit with the overall highest fracture properties represents the fracture mode of WHA and is shown in Figure 7. Most of the fracture surface is formed by transgranular matrix failure, W particle decohesion is also common. Very few fractured particles were observed on the fatigue fracture surface.

## 4. Conclusions

A fitting methodology to obtain parameters of the Hartmann–Schijve equation based on an inverse function fit in log–log coordinates was developed. This methodology was used to fit fatigue crack growth data of different RF-plasma-sprayed W- and Mo-based deposits obtained using resonance testing with a negative *R* ratio.

The used description of the fracture behavior covers both cyclic and static fracture properties over a wide range of loads and crack growth rates. It provides the extreme values approached by the crack driving force $K_{\max}$ at its minimum value defined by fatigue crack growth threshold $\Delta K_{\mathrm{thr}}$, and its maximum value at static fracture defined by fracture toughness $K_{\mathrm{IC}}$. This characterization enables two classes of materials to be separated and to assess the effect of powder pretreatment in the Ar-H${}_{2}$ reduction atmosphere.

### 4.1. Deposits of Inherently Brittle Materials (Mo, W, and the Mo-20W Composite)

1. Defects typical to plasma-sprayed materials such as intersplat interfaces and different types of porosity resulted in limited toughness values, from $K_{\mathrm{IC}}=7.3\;$MPa·m${}^{0.5}$ to $K_{\mathrm{IC}}=10.3\;$MPa·m${}^{0.5}$.
2. Fatigue crack growth thresholds from extremely low values of $\Delta K_{\mathrm{thr}}=0.5\;$MPa·m${}^{0.5}$ up to $\Delta K_{\mathrm{thr}}=5.4\;$MPa·m${}^{0.5}$ were observed, with highest values obtained for smaller particle size pretreated powders.
3. The Mo and W deposits have very similar fatigue crack growth resistance at the threshold value of $\frac{da}{dN}=10^{-10}\;$m·cycle${}^{-1}$ at $\Delta K_{\mathrm{th}}∼5.4\;$MPa·m${}^{0.5}$. Another W deposit from a coarse powder had much lower $\Delta K_{\mathrm{th}}=3.6\;$MPa·m${}^{0.5}$.

### 4.2. Tungsten Heavy Alloys

1. Adding Ni to the W powder led to a fully developed WHA structure that significantly increased both $\Delta K_{\mathrm{thr}}$ and $\Delta K_{\mathrm{IC}}$ values. This increase is caused by elimination of most of the intersplat interface imperfections by liquid state diffusion processes that take place during the deposition and by forming a ductile Ni-based matrix binder phase.
2. The saturated solid solution of W in Ni represents a ductile binder phase with a limited fatigue crack growth threshold value, as demonstrated through the W-65Ni deposit with very low $\Delta K_{\mathrm{thr}}∼2.4\;$MPa·m${}^{0.5}$.
3. The W particles from untreated powder in W-10Ni* deposits increase the threshold value $\Delta K_{\mathrm{thr}}$ significantly to $\Delta K_{\mathrm{thr}}\geq 11.5$ MPa·m${}^{0.5}$.
4. The use of pre-treated W powders further improves the strength of the reinforcement W particles and this in turn increases the fracture toughness of the deposit by a factor of three.

The suggested characterization methodology of FCGR curves provides a straightforward mathematical description of numerical FCGR data that can be used to describe the FCGR behavior of materials. The observed difference of $\Delta K_{\mathrm{th}}$ (at $\frac{da}{dN}=10^{-10}$ m·cycle${}^{-1}$) and $\Delta K_{\mathrm{thr}}$ (asymptotic value) indicates the importance of asymptotic description of the FCGR curve and justifies FCGR testing at very low $\frac{da}{dN}<10^{-10}$ m·cycle${}^{-1}$. The documented Matlab code of the fitting routines is available as a supplementary file to this paper.

## Figures and Tables

**Table 1:** Used feedstock powder information. The $D_{10}$ and $D_{90}$ represent particle size volumetric quantiles.

Powder	Manufacturer	Powder Designation	$D_{10}$ (µm)	$D_{90}$ (µm)	Type
Mo	Tekna		15	45	spheroidized
W${}_{1}$	GTP	MP65S	10	35	agglomerated-sintered
W${}_{2}$	Osram	MP75	15	45	agglomerated-sintered
Ni	H.C. Starck	Amperit 176.068	15	45	gas atomized

**Table 2:** Most important RF-ICP deposition parameters of the investigated materials. The nominal powder blend compositions are given in wt.%. Feedstocks denoted “(pre)” were pre-treated in reductive atmosphere before their spraying.

Material	Ref	Powder	Substrate	Scanning Speed (mm/s)	Stand-Off Distance (mm)	Carrier Gas	Carrier Gas (slpm)	Powder Feed Rate (g/min)
Mo		Mo (pre)	strip	1	90	Ar	8	12
Mo-20W		W${}_{1}$ + Mo (pre)	strip	1	90	He	8	12
W (fine-low)	[19]	W${}_{1}$	disc	0.7–1.2	70	He	8	2.3
W (fine-high)	[19]	W${}_{1}$	disc	0.7–1.2	70	He	8	3.7
W (coarse)	[9]	W${}_{2}$	disc	0.9–2.6	70	Ar	5	7.2
W		W${}_{1}$ (pre)	strip	1	90	He	8	12
W-10Ni*	[15]	W${}_{1}$ + Ni	strip	1	90	Ar	8	12
W-10Ni		W${}_{1}$ + Ni (pre)	strip	1	90	Ar	8	12
W-20Ni		W${}_{1}$ + Ni (pre)	strip	1	90	Ar	8	12
W-65Ni		W${}_{1}$ + Ni (pre)	strip	1	90	Ar	8	12

**Table 3:** Mechanical properties of the materials in tension (elastic modulus $E_{t}$, yield strength $R_{p}02t$, tensile strength $R_{m}$, and ductility A5) as well as fracture mechanical properties of the investigated refractory RF-ICP deposits and two powder metallurgy references.

Material	$K_{\mathrm{th}}$ (MPa·m${}^{0.5}$)	$K_{\mathrm{thr}}$ (MPa·m${}^{0.5}$)	$K_{\mathrm{IC}}$ (MPa·m${}^{0.5}$)	*D*	*p*	$E_{t}$ (GPa)	$R_{p}02t$ (MPa)	$R_{m}$ (MPa)	A5 $\left(10^{-3}\right)$
RF Mo	5.4	3.3	10.7	$3.12\times 10^{-14}$	7.65	279 ± 1	156 ± 21	393 ± 68	32.0 ± 4
RF Mo-20W	5.4	3.4	11.2	$6.89\times 10^{-17}$	13.58	269 ± 9	n.a.	100 ± 1	0.4
RF W (fine-low)	5.5	5.4	7.7	$2.16\times 10^{-08}$	2.41	211 ± 12	n.a.	290 ± 63	1.6 ± 0.2
RF W (fine-high)	5.5	5.4	7.3	$1.17\times 10^{-08}$	3.09	143 ± 20	n.a.	227 ± 33	1.6 ± 0.4
RF W (coarse)	3.6	0.5	10.3	$7.73\times 10^{-16}$	8.74	107 ± 0	n.a.	49 ± 7	1 ± 0.3
RF W	5.4	5.2	10.0	$1.37\times 10^{-09}$	2.97	309	n.a.	279	1.1
RF W-10Ni*	15.1	14.5	23.0	$9.79\times 10^{-11}$	1.54	342 ± 0	728 ± 36	751 ± 30	2.4 ± 1.5
RF W-10Ni	13.8	11.5	70.6	$4.47\times 10^{-12}$	3.24	362	681	1032	>6.7
RF W-20Ni	15.6	13.0	138.9	$1.30\times 10^{-12}$	4.15	339 ± 31	691 ± 15	1102 ± 120	>6.9 ± 0.3
RF W-65Ni	8.3	2.4	160.3	$9.43\times 10^{-16}$	6.47	234	477	630	>7.1
PM W-6Ni-3Co (SQ)	15.4	12.7	188.0	$1.83\times 10^{-12}$	3.84	306 ± 3	684 ± 2	980 ± 9	>6.0
PM W-6Ni-3Co (FA)	23.6	14.8	70.0	$1.70\times 10^{-16}$	5.57	321 ± 11	1596 ± 31	1693 ± 12	>6.0

## Data Availability

The FCGR data analyzed in this study as well as the Matlab routine to fit the H-S equation were made freely available at Cesnet repository (https://owncloud.cesnet.cz/index.php/s/EyhbscBYEowSXSG, accessed on 17 February 2023).

## Abbreviations

The following abbreviations are used in this manuscript:

FCGR	fatigue crack growth rate
SEM	scanning electron microscope
ECCI	electron channeling contrast imaging
H-S	Hartmann–Schijve
RF-ICP	radio frequency inductively coupled plasma
